# Capecitabine in combination with oxaliplatin and bevacizumab (AXELOX) as 1st line treatment for fit and vulnerable elderly patients (aged >70 years) with metastatic colorectal cancer (mCRC): a multicenter phase II study of the Hellenic Oncology Research Group (HORG)

**DOI:** 10.1186/1471-2407-14-277

**Published:** 2014-04-22

**Authors:** Lambros Vamvakas, Alexios Matikas, Athanasios Karampeazis, Dora Hatzidaki, Stelios Kakolyris, Charalampos Christophylakis, Ioannis Boukovinas, Aris Polyzos, Vassilis Georgoulias, John Souglakos

**Affiliations:** 1 Hellenic Oncology Research Group (HORG), 55 Lomvardou str, 11470 Athens, Greece

**Keywords:** XELOX, Capecitabine, Oxaliplatin, Bevacizumab, mCRC, Colorectal cancer, Elderly patients

## Abstract

**Background:**

Colorectal cancer (CRC) is a disease of the elderly. However, geriatric patients are often excluded from clinical trials. The combination of capecitabine, oxaliplatin and bevacizumab (XELOX/BEV) has not been assessed in an elderly population.

**Methods:**

We conducted a phase II study of XELOX plus bevacizumab combination as first line treatment in elderly patients with metastatic CRC. Treatment consisted of capecitabine 750 mg/m^2^ twice a day during days 1–7, oxaliplatin 85 mg/m^2^ and bevacizumab 5 mg/kg on day 1. Treatment was repeated every 14 days. The primary endpoint was overall response rate.

**Results:**

In the 48 enrolled patients response rate according was 46.8% (95% CI: 32.54%–61.07%), while 13 patients had stable disease, for an overall disease control rate of 74.4% (95% CI: 57.8–91.2). Progression free survival was 7.9 months (95% CI: 5.9–9.8 months) and the median overall survival 20.1 months (95% CI: 15.6–25.7 months). Response rate and progression free survival has been correlated with baseline albumin and haemoglobin levels. There was one treatment-related death. Grade 3–4 toxicities were asthenia (4.2%), neurotoxicity (2.1%) and diarrhea 6.3%).

**Conclusions:**

The combination of capecitabine, oxaliplatin and bevacizumab is an effective and safe combination for the treatment of elderly patients with metastatic CRC.

**Trial registration:**

Clinical trials NCT01024504, 26 November 2010.

## Background

Colorectal cancer is a leading cause of cancer related death in developed countries [1]. Considering the fact that approximately 60% of cases are diagnosed in patients 65 years or older and also the steadily growing elderly population in the developed world, it is expected that the total number of CRC patients over 65 will rise significantly in the future and CRC will become a major health issue in geriatrics [2].

There are numerous challenges regarding the treatment of elderly patients with metastatic CRC (mCRC). Normal aging results in diminished organ function and reserves; liver and kidney function declines thus altering drug metabolism, the risk of coronary artery disease and arterial thromboembolic events is increased, bone marrow reserves are limited and polypharmacy and comorbidities must be taken into account when treatment is planned [3,4]. A number of tools has been developed that assess functional status and predict chemotherapy toxicity and risk of death in elderly patients [5,6]. One of the most widely used tools is the Comprehensive Geriatric Assessment (CGA) which includes functional, medical, psychosocial and nutritional questionnaires [7].

The combination of oxaliplatin and fluorouracil (FOLFOX) has been consistently shown to be effective and well tolerated among fit elderly patients with mCRC in prospective randomized trials and pooled analyses [8-10]. Moreover, capecitabine combined with oxaliplatin (XELOX) has been shown to be non-inferior to FOLFOX, albeit with slightly increased toxicity [10,11]. The addition of the monoclonal antibody bevacizumab, which targets the Vascular Endothelial Growth Factor-A (VEGF-A) to chemotherapy, including XELOX [12], is considered standard practice when treating younger mCRC patients [13]. However, when given to elderly patients there is a concern regarding an increased risk for arterial thromboembolic events [14]. The combination of bevacizumab and capecitabine has been shown to be effective in this setting, resulting in an increased progression free survival (PFS) in an elderly – specific phase III trial that was recently published [15]. The combination of bevacizumab with two active chemotherapeutics has not been prospectively assessed in an exclusively elderly population.

The aim of the current study is to examine the efficacy and safety of the combination of capecitabine, oxaliplatin and bevacizumab (XELOX/BEV) among elderly patients with mCRC and to identify predictive and prognostic factors derived from multidimensional geriatric evaluations that influence response to treatment, progression free survival (PFS), overall survival (OS) and toxicity.

## Methods

### Patients

Eligible patients had histologically confirmed unresectable locally advanced or metastatic colorectal cancer, were ≥ 70 years old with an Eastern Cooperative Oncology Group Performance Status (ECOG PS) of 0 to 2 and had not received any prior chemotherapy or biologic agent for metastatic colorectal cancer; adjuvant chemotherapy with a fluoropyrimidine was allowed if 6 months or more had elapsed since its completion. Other eligibility criteria included measurable disease by Response Evaluation Criteria in Solid Tumors (RECIST) 1.0; [16] adequate bone marrow, liver and kidney function defined as Glomerular Filtration Rate (GFR) as calculated using the Cockcroft-Gault formula ≥30 ml/min; absence of brain metastases; a life expectancy of at least three months at enrollment; frail patients as determined by the Comprehensive Geriatric Assessment (CGA) were excluded [17]. Other exclusion criteria were the daily use of aspirin (>325 mg per day), other non-steroidal anti-inflammatory drugs or anticoagulants at a therapeutic dose; and significant cardiovascular disease such as uncontrolled hypertension, coronary heart disease or previous stroke. The study has been approved by the ethics and scientific committees of the participating centers and all patients gave their written informed consent in order to enter the study (Scientific Committees and Committees for Bioethics and Medical Ethics, University Hospital of Herakleion; University General Hospital of Alexandroupolis; IASO General Hospital of Athens; Air Forces Military Hospital of Athens; Laikon General Hospital; State General Hospital of Larissa; Theagenion Anticancer Hospital of Thessaloniki).

### Treatment

We used a modified XELOX regimen, administered capecitabine in one week on one week of schedule, since this is associated with less gastrointestinal adverse events and hand and foot syndrome [18]. Capecitabine was given orally at a dose of 750 mg/m^2^ twice a day during days 1–7, a regimen that was found to be effective at a phase II study previously conducted by our group, albeit with a lower dose due to increased toxicity concerns when combined with both oxaliplatin and bevacizumab [18]. The dose was lowered to 625 mg/m^2^ twice a day if the Glomerular Filtration Rate (GFR), as calculated using the Cockcroft-Gault formula, was ≤60 ml/min. Oxaliplatin was given intravenously at a dose of 85 mg/m^2^ over 240 minutes on day 1. Bevacizumab was given intravenously at a dose of 5 mg/kg over 60–90 minutes according to investigator discretion, on day 1. Treatment was administered every two weeks for a total of 12 cycles of chemotherapy except for cases of disease progression, unacceptable toxicity or consent withdrawal. Treatment was postponed for 1 week if on day 1 of each cycle neutrophils were <1500/μL, platelets < 100000/μL and if there were other > grade 2 toxicities. If a patient had not recovered after delaying treatment for 2 weeks, he was removed from the study.

The prophylactic use of Granulocyte Colony Stimulating Factor (G-CSF) was not allowed except for the secondary prevention of neutropenic fever. Erythropoiesis stimulating agents were allowed in cases of grade 2 or worse anemia and were discontinued when haemoglobin levels exceeded 12gr/dL. Pre-specified dose modifications were allowed for capecitabine and oxaliplatin for grade 3–4 haemotological adverse events and grade 2–4 non-haematological adverse events. The dose of bevacizumab was reduced by 50% in cases of uncontrollable arterial hypertension despite antihypertensive treatment and it was discontinued in cases of severe haemorrhagic events.

### Patient evaluation

Pre-treatment evaluation included a detailed medical history and physical examination, a complete blood cell count (CBC) with differential and platelet count, blood chemistry, serum levels of carcinoembryonic antigen (CEA) and computed tomography scans (CT) of the chest and abdomen. Patients were clinically assessed before each cycle, CBC was performed weekly and blood chemistry every two weeks before treatment administration.

Responses were evaluated with CT scans of the chest and abdomen and by measuring CEA levels every three months. Bone scintigraphy and brain CT were performed based on clinical indication. The Response Evaluation Criteria in Solid Tumors (RECIST) were used to assess tumor responses. The duration of response was measured from the first documentation of response to disease progression. The progression free survival (PFS) was determined by the interval between the initiation of treatment and the date when disease progression was first documented or the date of death from any cause. Overall survival (OS) was measured from the date of treatment initiation to the date of death. The follow up time was measured from the day of first treatment administration to the time of the present analysis (for patients still alive) or death for deceased patients.

Toxicities were graded according to the Common Terminology Criteria for Adverse Events (CTCAE) 3.0. All patients underwent a Comprehensive Geriatric Assessment (CGA) at study enrollment, after the 6th and after the 12th cycle. The CGA includes questionnaires of daily (ADL) and instrumental (IADL) activities, the assessment of performance status, nutritional, mental (Mini Mental State Examination) and emotional status (Geriatric Depression Scale), as well as the evaluation for the presence of geriatric syndromes, the Charlson’s comorbidities index and the evaluation of concomitant medications. Patients were classified as fit if they were independent during ADL and IADL and there were no geriatric syndromes or comorbidities. Patients were classified as vulnerable if they were dependent on some IADL but not ADL, there were minor comorbidities but no geriatric syndromes.

### Statistical analysis

This multicenter single-arm prospective phase II study evaluated the efficacy and safety of the XELOX/BEV combination as front line treatment for elderly patients with metastatic colorectal cancer. The sample size calculation was conducted according to an optimal Simon two-step design testing: The null hypothesis was that the ORR is ≤ 30% versus the alternative hypothesis that the ORR is ≥ 40% (α = 0.05, power 80%). An interim analysis took place on the first 15 patients and as the number of responders was at least 6, then 31 additional patients were planned to be enrolled for a total of 46 patients.

The analysis of the primary endpoint was performed for the intent-to-treat population, defined as all patients who received at least one treatment cycle in the study. Patients who withdrew without a response assessment were classed as non-responders in the analysis. Chi-square test was used for the association of response with other dichotomous variables (patients’ age, sex, tumor stage, etc.). Binary logistic regression was carried out in order to evaluate which of the significant factors at the chi-square analysis had a significant influence on response. Cox’s proportional hazards multivariate analysis was used to evaluate which of the significant factors at the univariate analysis had a significant influence on PFS and OS. Statistical significance was set at *p* = 0.05.

## Results

### Patient characteristics

Between 1/3/2008 and 26/3/2012 48 patients from 11 centers were enrolled. The patients’ demographic and clinical characteristics are summarized on Table 1. The median age was 76 years (range: 70 – 86) and 50% (n = 24) of the patients were female. The median number of metastatic sites was 2. According to the CGA assessment at baseline, 52% of the patients were classified as fit and 48% as vulnerable. The results of multiple geriatric assessment tools such as the Geriatric Depression Scale, Mini Mental State Examination, Instrumental Activities of Daily Life and Charlson Comorbidity Index are shown on Table 1.

**Table 1:** Patient demographic and clinical characteristics

	**Number of patients (n = 48)**	**(%)**
**Age**		
Median	76	
Min-Max	70–86	
**Sex**		
Male	24	50.0
Female	24	50.0
**Performance status (WHO)**		
0	11	22.9
1	33	68.8
2	4	8.3
**Previous Treatment**		
Surgery	36	75.0
Adjuvant chemotherapy	9	18.8
Adjuvant radiotherapy	4	8.3
**Disease Sites**		
Lymph nodes	15	31.3
Liver	36	75.0
Lung	17	35.4
Other	11	23
**Number of Metastatic Sites**		
1	22	45.8
2	14	29.2
3	10	20.8
4	1	2.1
**CGA (n = 48)**		
Fit	25	52.0
Vulnerable	23	48.0
**GDS (n = 30)**		
≤5	22	73.3
>5	8	26.7
Median	4	
Range	1–12	
**MMSE (n = 32)**		
<24	4	12.5
≥24	28	27.5
Median	27	
Range	3–30	
**IADL (n = 37)**		
<7	10	73.3
7–8	27	26.7
Median	8.0	
Range	3–8	
**Charlson Comorbidity Index**		
Median	1	
Range	0–2	
**Body Mass Index**		
Median	27.75	
Range	18.0–35.0	
**Haemoglobin (gr/dL)**		
Median	11.8	
Range	9.7–16.4	
**Albumin (gr/dL)**		
Median	3.9	
Range	2.6–4.8	
**Medications**		
Median	1	
Range	0–4	

### Compliance with the treatment

A total of 409 chemotherapy cycles were administered (median 10, range: 1–16). A total of 41 cycles (10%) were delayed with a median delay of 9 days (range, 5–66 days). The reason for treatment delay was haematologic (n = 9 cycles), non-haematologic (n = 5 cycles) toxicity or both (n = 1 cycle), while in 26 cycles the delay was related with late admission or response evaluation. Dose reduction was required in 18 cycles (4.4%) because of haematological (n = 2 cycles) and non-haematological (n = 12 cycles) as well as for non-toxicity related reasons (n = 4 cycles). Three patients discontinued treatment because of treatment-related toxicity and two patients withdrew their consent. The mean dose intensity was 4700 mg/m^2^/week for capecitabine corresponding to the 89.5% of the preplanned protocol dose, 40 mg/m^2^/week for oxaliplatin corresponding to the 94.5% of the preplanned protocol dose and 2.3 mg/kg/week for bevacizumab corresponding to the 92% of preplanned dose. G-CSF was not used since none of the patients developed febrile neutropenia, while erethropoetin was administered in two patients (4%) for the treatment of grade II anemia. Twenty-three (47.9%) patients completed the treatment as per protocol, 1 patient was lost to follow-up, 2 patients underwent definitive liver metastasectomy, and 1 patient discontinued treatment because of an episode of acute cholecystitis.

### Treatment efficacy

Forty-seven patients were evaluable for response to treatment. The study met its primary end point since in an intension to treat analysis, there were four (8.5%) complete [19] and 18 (38.3%) partial responses (PR), for an overall response rate of 46.8% (95% CI: 32.5%–61.1%). Thirteen (27.7%) additional patients had stable disease (SD), for a disease control rate (CR + PR + SD) of 74.4%. The median response duration was 7.1 months (range, 1.2–21.0). After a median follow-up of 15.6 months (range: 0.1–45.4 months), the median progression free survival was 7.9 months (95% CI: 5.9–9.8 months) and the median overall survival 20.1 months (95% CI: 15.6–25.7 months). The probability of 1- and 2-year survival rate were 76.7% and 42.1%, respectively.

**Figure 1:**
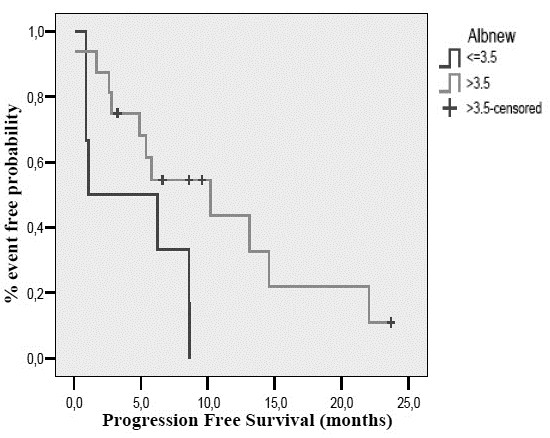
Progression free survival according to baseline albumin levels.

**Figure 2:**
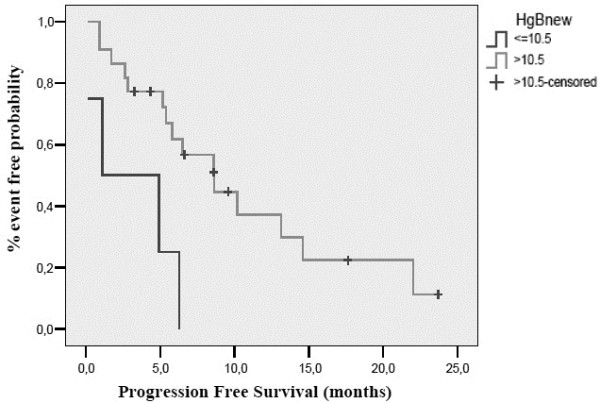
Progression free survival according to baseline haemoglobin levels.

**Figure 3:**
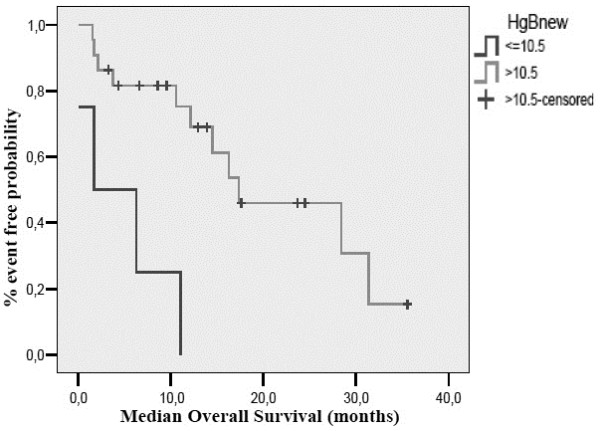
Overall survival according to baseline haemoglobin levels.

On pre-planned subgroup analysis, response rate was found to be affected by baseline albumin levels. Of the 22 patients that had a baseline albumin measurement, all responses were observed in patients with an albumin levels >3.5 gr/dL (ORR 50%) compared to patients with an albumin level <3.5 gr/dL (ORR%; p = 0.030). Response to treatment was not affected by status according to comprehensive geriatric assessment (p = 0.634), IADL score (p = 0.920), MMSE score (p = 0.285), GDS score (p = 0.151), haemoglobin levels (p = 0.086) or body mass index (BMI, p = 0.457). PFS was affected by baseline albumin (10.2 months for albumin ≥3.5 gr/dL versus 1.1 months for albumin <3.5 gr/dL, p = 0.038) and baseline haemoglobin [20] levels (8.6 months for Hb ≥ 10.5 gr/dL versus 1.1 months for Hb < 10.5 gr/dL, p = 0.007) (Figures 1 and 2). CGA (p = 0.272), IADL (p = 0.805), MMSE (p = 0.413), GDS (p = 0.088) and BMI (p = 0.551) did not affect PFS. Finally, OS was only affected by Hb levels (17.4 months for Hb ≥ 10.5 gr/dL versus 1.1 months for Hb < 10.5 gr/dl, p = 0.001) (Figure 3). OS according to CGA status was 20.7 months for fit patients versus 17.4 months for vulnerable patients, a difference that could not reach statistical significance. These results were independent of patients’ age (71–80 vs. 81 or higher), PS, number of organs involved and Kohne prognostic index.

### Toxicity

Treatment related adverse events are summarized on Table 2. There was one toxic death due to acute respiratory distress syndrome attributed to oxaliplatin. The most common adverse event was grade 1–2 anemia (79.1%), grade 1–3 asthenia (54.2%; 4.2% grade 3), grade 1–3 neurotoxicity (37.5%) with one patient developing grade 3 neurotoxicity and grade 1–3 diarrhea (29.2%) with grade 3 occurring in three patients. There was only one patient who developed grade 4 arterial hypertension leading to bevacizumab dose reduction. There were no thromboembolic episodes. There were only 3 episodes of grade 3 neutropenia (6.3%) but there was no case of neutropenic fever. The incidence of adverse events was not influenced by the patient status (fit vs vulnerable).

**Table 2:** Treatment related adverse events

	**All grades**		**Grades III-IV**	
	**N**	**%**	**N**	**%**
Leukopenia	12	25.1	-	**-**
Neutropenia	11	23.0	3	6.3
Anemia	38	79.1	-	**-**
Thrombocytopenia	18	37.5	-	**-**
Nausea	11	23.0	2	4.2
Vomiting	9	18.8	2	4.2
Diarrhea	13	27.2	3	6.3
Mucositis	3	6.3	-	**-**
Dysgefsia	1	2.1		
Constipation	9	18.8	1	2.1
Abdominal pain	4	8.3		
Non cardiac chest pain	1	2.1		
Neurotoxicity	18	37.5	1	2.1
Seizures	1	2.1		
Headache	3	6.3		
Allergic reaction	1	2.1	1	**-**
Asthenia	264	54.2	2	4.2
Anorexia	5	10.4		
Cutaneous toxicity	1	2.1	-	**-**
Fever	2	4.2	-	**-**
Hemorrhage	1	2.1	-	**-**
Hypertension	3	6.3	1	2.1
				.1
Hand foot syndrome	1	2.1		

## Discussion

The treatment of elderly patients with mCRC is still considered to be a debatable issue. Despite the fact that the majority of patients are diagnosed in advanced age, elderly patients under-represented in randomized clinical trials. In addition, the vast majority of elderly patients included in randomized trials are usually selected and only fit elderly patients are finally enrolled. Due to this fact, no definitive conclusion can be drawn regarding the treatment of this population group with mCRC.

The current study describes the results of a chemotherapy doublet plus bevacizumab in elderly patients selected upon CGA. Our results show that the triple combination of XELOX/BEV is effective and well tolerated among fit and vulnerable elderly patients with mCRC. The response rate, PFS and OS compares well to results in younger populations reported in the literature.

Our study has several strengths. First of all, to our knowledge this is the first study to prospectively assess a three drugs combination specifically in an elderly-only population. Designing geriatric-specific chemotherapy trials has been shown to allow for the prediction of treatment related toxicities [19]. This is further supported by our study where the rates of reported adverse events were very low. Also, compliance was excellent with very low rates of dose reduction or cycle postponement. Other strengths of our study include the systematic use of comprehensive geriatric assessments and the homogenous study population, which allowed for careful dose tailoring which led to high response rates with minimal toxicity.

On the other hand, our study suffers from several weaknesses. It is a phase II study with a small number of patients. As a result, we were unable to identify predictive and prognostic factors derived from the Comprehensive Geriatric Assessments. Also, our study population was limited only to fit and vulnerable elderly patients, so our results may not be representative in frail patients with mCRC.

Previous studies have established the efficacy and safety of combination chemotherapy in elderly patients [8-11]. The addition of bevacizumab to chemotherapy in geriatric populations has also been shown to be effective in observational cohort studies, subgroup analyses and pooled analyses of cohorts of other randomized trials [20-23]. Recently, the results of the first randomized Phase III study of capecitabine plus bevacizumab exclusively in elderly patients were published (AVEX trial: bevacizumab plus capecitabine versus capecitabine in elderly patients with previously untreated metastatic colorectal cancer) [15]. Taking into account the inherent hazards of cross-study comparisons, OS was similar between the AVEX trial and our study. However, response rates were higher with the triple drug combination and adverse events were lower. In addition, the patient population in the AVEX study was quite different since it was based on clinical judgment that the patient was not suitable for oxaliplatin or irinotecan-based doublets. It seems that the next logical step would be to directly compare these two regimens; until such data are available, modified XELOX/BEV might be an alternative option for patients requiring a relatively rapid and objective clinical response, such as in patients with potentially resectable disease or in those with rapidly progressive disease and/or with a need for symptoms relief.

## Conclusion

In conclusion, our data suggest that the combination of XELOX/BEV combination is an attractive and alternative regimen for fit and vulnerable elderly patients with mCRC, with high response rates and low toxicity. Also, the use of CGA at baseline may be considered as a tool capable to differentiate elderly patients who are fit enough to tolerate a more intensive treatment. Further evaluation in randomized trials is needed in order to confirm these observations.

## Competing interests

The authors declare that they have no competing interests.

## Authors’ contributions

LV has contributed in the acquisition of data and drafting the manuscript, AM has contributed in the acquisition of data and helped to draft the manuscript, AK has contributed in the acquisition of data, DH has contributed in the analysis and interpretation of data, SK has contributed in the acquisition of data, CC has contributed in the acquisition of data, IB has contributed in the acquisition of data, AP has contributed in the acquisition of data, VG participated in the design of the study and data interpretation and helped in drafting the manuscript, JS has contributed in study conception and design, analysis and interpretation of data and drafting the manuscript. All the authors gave their final approval of the version to be published.

## Pre-publication history

The pre-publication history for this paper can be accessed here:

http://www.biomedcentral.com/1471-2407/14/277/prepub
